# High prevalence of coexisting prehypertension and prediabetes among healthy adults in northern and northeastern China

**DOI:** 10.1186/1471-2458-11-794

**Published:** 2011-10-12

**Authors:** Jie Wu, Wen-hua Yan, Ling Qiu, Xin-qi Chen, Xiu-zhi Guo, Wei Wu, Liang-yu Xia, Xu-zhen Qin, Yan-hong Liu, Hai-tao Ding, Shao-mei Han, Cheng-li Xu, Guang-jin Zhu

**Affiliations:** 1 Department of Clinical Laboratory, Peking Union Medical College Hospital, Peking Union Medical College & Chinese Academy of Medical Science, Beijing 100730, China; 2 Department of Endocrinology, Chinese PLA General Hospital, Beijing 100853, China; 3 Department of Clinical Laboratory, The 2nd Affiliated Hospital of Harbin Medical University, Harbin 150001, China; 4 Department of Clinical Laboratory, Inner Mongolian People's Hospital, Hohhot, 010017, China; 5 Institute of Basic Medical Sciences, Chinese Academy of Medical Sciences & Peking Union Medical College, Beijing 100005, China

## Abstract

**Background:**

Prehypertension and prediabetes are major risk factors of cardiovascular disease, and their combined presence may result in more serious cardiovascular outcomes than expected with either prehypertension or prediabetes alone. The aim of the present study was to evaluate the prevalence of coexisting prehypertension and prediabetes, and the associated risk profiles in a Chinese population.

**Methods:**

A cross-sectional survey in a representative sample of 3,595 men and 4,593 women aged 18 years and older was performed between 2008 and 2010. Prehypertension and prediabetes were diagnosed using the guidelines from the Seventh Report of the Joint National Committee on prevention, detection, and treatment of high blood pressure and American Diabetes Association, respectively. Prehypertension was defined as a systolic blood pressure of 120-139 mmHg and/or diastolic blood pressure of 80-89 mmHg, and prediabetes was defined as a fasting blood glucose of 5.6-6.9 mmol/L.

**Results:**

The prevalence of coexisting prehypertension and prediabetes was 11.0%. Men had a higher prevalence of coexisting prehypertension and prediabetes than women (14.2% vs. 8.4%; *P *< 0.0001). This prevalence increased with age and body mass index, and was the lowest among Mongolian-Chinese (5.1%). A multivariate analysis showed that γ-glutamyltransferase and uric acid were significantly and positively correlated with body mass index, waist circumference, blood pressure, triglycerides, and total cholesterol, and negatively correlated with high density lipoprotein cholesterol in subjects with prehypertension and prediabetes.

**Conclusions:**

There is a large proportion of Chinese adults with coexisting prehypertension and prediabetes. Thus, there is a need for more efforts that implement public health programs that target the earlier stages of hypertension and diabetes.

## Background

Cardiovascular disease (CVD) is considered to be a major cause of death in most developed and developing countries [1]. Hypertension is a major risk factor of CVD. Even a slightly elevated blood pressure within the normal range is associated with cardiovascular morbidity and mortality [2]. Therefore, in 2003, the Seventh Report of the Joint National Committee on the Prevention, Detection, Evaluation, and Treatment of High Blood Pressure (JNC-7) introduced a new category: prehypertension (PreHTN), where systolic blood pressure (SBP) is from 120 to 139 mmHg, and/or diastolic blood pressure (DBP) is from 80 to 89 mmHg [3]. Individuals with PreHTN are more susceptible to developing true hypertension and coronary atherosclerosis [4]. Several national blood pressure surveys in the United States and other countries report that more than 30% of the general adult population has PreHTN [5,6]. Prediabetes (PreDM) is another important risk factor of CVD, which is associated with impaired fasting glucose (IFG) and/or glucose tolerance (IGT). According to the American Diabetes Association, PreDM is indicated by IFG, where serum glucose concentrations range from 5.6 mmol/L (100 mg/dl) to 6.9 mmol/L (125 mg/dl), as well as IGT, where serum glucose concentrations range from 7.8 mmol/L (140 mg/dl) to 11.1 mmol/L (199 mg/dl) 2 h after a 75 g oral glucose load [7]. IFG is associated with insulin resistance and an increased risk of cardiovascular pathology. Although less risky than IGT, IFG is coupled with a greater conversion from PreDM to overt diabetes (i.e. approximately 24% in less than three years) [8]. Recently published data on a large population-based sample of more than 45,000 people, aged 20 years and older, in China indicated that the prevalence of PreDM was 15.5% [9].

Given that the effects of certain clinical precursors, such as PreHTN and PreDM, on the cardiovascular system are often distinct, their combined presence in the same patient may result in more severe coronary artery disease than expected with either PreHTN or PreDM alone [10]. The probable CVD risk with PreHTN and PreDM, to some extent, is dependent on whether PreHTN leads to hypertension and PreDM leads to diabetes. Additionally, racial and ethnic differences are known to influence the prevalence and risks of PreHTN and PreDM [11,12]. Several large surveys have yielded prevalence estimates for either PreHTN or PreDM in China [9,13]. Nevertheless, there is little information available in the literature regarding the prevalence of coexisting PreHTN and PreDM (co-PreHTN and PreDM) among individuals located in areas inhabited by Chinese ethnic minorities. Furthermore, to the best of our knowledge, there have been no population-based, cross-sectional studies that have assessed the epidemiology of co-PreHTN and PreDM among healthy Chinese adults. Thus, the aim of the present study was to evaluate the prevalence of co-PreHTN and PreDM, along with their associated risks, in populations located in northern and northeastern China.

## Methods

### Study population

A population-based, cross-sectional survey of the Chinese Physiological Constant and Health Condition (CPCHC) was conducted between 2008 and 2010. Representative samples of the general Chinese population, aged 18 years and older, from the Hei Longjiang Province and Inner Mongolian Autonomous Region in mainland China were determined according to a random, multistage cluster sampling scheme, allowing for good prevalence estimates of the Chinese population. Two urban and two rural areas were selected from each province. Written informed consent was obtained from each participant prior to data collection. The protocol was approved by the Institutional Review Board of the Institute of Basic Medical Sciences, Chinese Academy of Medical Sciences. Trained medical personnel collected information on risk factors via questionnaires (e.g. demographic, socioeconomic, and health-related information), and obtaining anthropometric measurements and blood samples for biochemical assessments.

### Inclusions and exclusions

**Figure 1:**
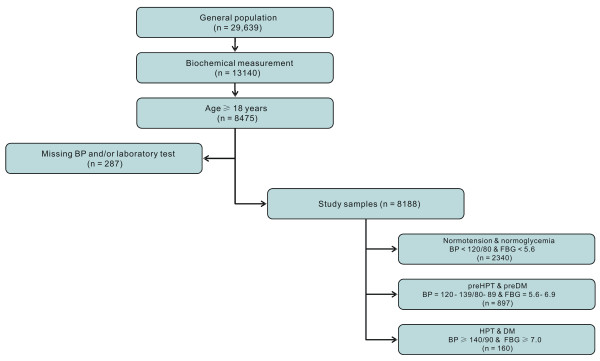
**A schematic used for screening and inclusion of the study sample**. A total of 29,639 disease-free and healthy individuals were recruited between 2008 and 2010, and 13,140 individuals had biochemistry measurements collected. Of the 8,475 adults aged ≥18 years, 287 participants had missing data on BP and/or laboratory tests, and as result were excluded. The final sample size was 8,188, which consisted of 2,340 individuals with both normotension and normoglycemia, 897 individuals with coexisting prehypertension and prediabetes, and 160 individuals with both hypertension and diabetes. Abbreviations: BP, blood pressure; FBG, fasting blood glucose; PreHTN, prehypertension; PreDM, prediabetes; HTN, hypertension; DM, diabetes mellitus.

The 2008-2010 CPCHC samples included 29,639 apparently healthy participants. Those who suffered from systemic disease involving diabetes mellitus, hypertension or other cardiovascular, renal, gastro-intestinal, pulmonary disease or cancer were excluded. Moreover, participants taking any medication known to affect carbohydrate and lipid metabolism were also excluded. A schematic of the screening process is presented in Figure 1. Of the total number of participants, 44.3% (n = 13,140) were selected randomly to complete blood testing. Of 8,475 adults, aged ≥18 years, 287 (3.4%) had missing data on blood pressure (BP) and/or laboratory tests. Therefore, the final sample size of disease-free healthy adults was 8,188 (3,595 men and 4,593 women), of which 5,788 were Han Chinese, 936 were Korean-Chinese, 1,237 were Mongolian-Chinese, and 227 were of other ethnicity. Their age distribution was as follows: 3,214 were aged 18-39 years, 1,762 were aged 40-49 years, 1,580 were aged 50-59 years, and 1,632 were aged > 60 years.

### Data collection and anthropometry

Epidemiological data were collected on all subjects via a standard questionnaire, which included demographic characteristics (i.e. age, gender, and ethnicity), socioeconomic data (i.e. educational level, marital status, and occupation), past history, and lifestyle risk factors. Smoking status was classified as non-smokers, current smokers (i.e. daily smoking regardless of the amount and type), and ex-smokers. Alcohol drinking status was defined as non-drinkers, current drinkers (frequent consumption of alcohol regardless of the amount and type), and ex-drinkers.

Body weight was measured to the nearest 0.1 kg on a calibrated beam scale and height was measured barefoot in triplicate using a wall-mounted stadiometer to the nearest 0.1 cm. Body mass index (BMI; an index of overall obesity) was calculated as body weight (in kilograms) divided by height (in meters squared). BMI was categorized according to the World Health Organization criteria, where a BMI of < 25 kg/m^2 ^is considered normal, a BMI between 25 and 29 kg/m^2 ^is considered overweight, and a BMI ≥30 kg/m^2 ^is considered obese [14]. Waist circumference (WC; a surrogate marker for central adiposity) was measured midway between the lower rib margin and the iliac crest at the end of a gentle expiration.

BP was measured following a resting period of at least 10 min using an electronic sphygmomanometer (OMRON, HEM-7000). The participant's arm was placed at the level of the heart, and BP was measured three times. The averages of the three measurements were used. If a subject was hypertensive, then a review was performed by doctors to exclude secondary hypertension.

### Laboratory measurements

All procedures were performed following a 12-h overnight fast. Blood was drawn from the antecubital vein of the right arm. Serum γ-glutamyltransferase (GGT; a sensitive marker of alcohol intake and hepatic inflammation) and uric acid (UA; a marker of inflammation and metabolic syndrome), both of which play important roles in the development of cardiovascular events, were assayed with an Olympus AU2700 Automatic Biochemical Analyzer and Olympus agent (Olympus, Tokyo, Japan). Fasting blood glucose and lipid profiles, including total cholesterol (TC), triglycerides (TG), high-density lipoprotein cholesterol (HDL-C), and low-density lipoprotein cholesterol (LDL-C) were also assessed. The biochemical laboratories participating in the survey followed the same internal quality control program that was standardized by the Peking Union Medical College Hospital.

### Diagnosis and classification of diabetes and hypertension

Diagnosis of PreHTN was based on the criteria in the JNC-7 report [3]. Specifically, PreHTN was defined as a SBP of 120-139 mmHg and/or a DBP of 80-89 mmHg, whereas normotension was defined as a SBP < 120 mmHg and a DBP < 80 mmHg, and hypertension was defined as a SBP ≥140 mmHg and/or a DBP ≥90 mmHg.

Diagnosis of PreDM was based on the criteria of the American Diabetes Association [7]. Pre-DM was defined as fasting blood glucose (FBG) levels from 100 mg/dl (5.6 mmol/L) to 125 mg/dl (6.9 mmol/L)], which indicated IFG, and/or 2-h postprandial blood glucose (PBG) levels from 140 mg/dl (7.8 mmol/L) to 199 mg/dl (11.1 mmol/L) following a 75 g oral glucose load, which indicated IGT. Individuals with FBG < 5.6 mmol/L were considered normal, whereas individuals with FBG ≥7.0 or 2-h PBG ≥11.1 were diagnosed with diabetes. Fasting was defined as no caloric intake for at least 8 h.

### Statistical analysis

Data were entered and documented on EpiData 3.1 software (The EpiData Association, Odense, Denmark). Datasets were transferred into an SPSS compatible format. Data are presented as counts and percentages ± standard errors (SE) for categorical variables, and means ± standard deviation (SD) for continuous variables with a normal distribution. Comparisons between groups were made using an analysis of covariance. Medians and interquartile ranges of GGT and TG were calculated due to their abnormal distributions, and comparisons between these groups were made using the Wilcoxon rank sum test. Prevalence (%) indicates the percentage of healthy Chinese men and women with a condition at the time of data collection, and means indicate the average value of a characteristic in healthy Chinese adults. Correlation analyses were performed using either the Pearson or Spearman correlations. Statistical analyses were performed on the statistical software, SPSS version 13.0 (SPSS Inc., Chicago, Illinois, USA). All tests for statistical significance were two-tailed, and considered significant when *P *< 0.05.

## Results

### Epidemiology

Of the 8,188 disease-free adults, 897 (11.0 ± 0.3%) had co-PreHTN and PreDM, 160 (2.0 ± 0.2%) had concurrent hypertension and diabetes mellitus, 2,340 (28.6 ± 0.5%) had both normotension and normoglycemia, and the remaining 4,791 had other combinations of resting BP and fasting serum glucose concentrations as shown in Table 1.

**Table 1:** Number of disease-free adults categorized by resting blood pressure and serum glucose levels

		Resting blood pressure		
	
Fasting blood glucose	Normal	Prehypertension	Hypertension (SBP &DBP)	Total
	(< 120/80 mmHg)	(120-139/80-89 mmHg)	(≥ 140/90 mmHg)	
Normal (< 5.6 mmol/L)	2340	2625	1057	6022
Prediabetes (5.6 - 6.9 mmol/L)	375	897	498	1770
Diabetes (≥ 7.0 mmol/L)	40	196	160	396
Total	2755	3718	1715	8188

Sample population was classified by blood pressure and glucose status.

The prevalence of co-PreHTN and PreDM was higher in the Hei Longjiang province compared to the Inner Mongolian Autonomous Region (13.3% vs. 8.8%, *P *< 0.0001), as shown in Table 2. Men had a higher prevalence of co-PreHTN and PreDM than women (14.2% vs. 8.4%, *P *< 0.0001). The prevalence increased with age (i.e. 6.6% for those 18-39 years old, 11.6% for those 40-49 years old, 14.0% for those 50-59 years old, and 15.9% for those > 60 years old) and BMI (i.e. 6.0% for those with a BMI < 18.5 kg/m^2^, 9.1% for those with a BMI between 18.5 and 24.9 kg/m^2^, 14.7% for those with a BMI between 25 and 29.9 kg/m^2^, and 12.5% for those with a BMI ≥30 kg/m^2^). Mongolian-Chinese (5.1%) had the lowest prevalence of co-PreHTN and PreDM, whereas both the Han-Chinese (12.0%) and Korean-Chinese (11.6%) demonstrated a two-fold greater risk of co-PreHTN and PreDM than the Mongolian-Chinese. Furthermore, those with a lower educational level, such as primary or secondary education, tended to have a higher prevalence of co-PreHTN and PreDM. Co-PreHTN and PreDM participants were more likely to be married, ex-smoking, and current alcohol drinkers (*P *< 0.0001).

**Table 2:** Prevalence (%) of healthy Chinese adults with either normotension and normoglycemia, prehypertension and prediabetes, or hypertension and diabetes mellitus

	Normotension and Normoglycemia		PreHTN and PreDM		HPT and DM	
	
	N	Prevalence	N	Prevalence	N	Prevalence
		(%)		(%)		(%)
Overall (2008-2010)	2340	28.6 ± 0.5	897	11.0 ± 0.3	160	2.0 ± 0.2
Survey area						
Hei Longjiang province	1137	29.3 ± 0.6	519	13.3 ± 0.5	97	2.5 ± 0.3
Inner Mongolian Autonomous Region	1203	27.9 ± 0.7	378	8.8 ± 0.4	63	1.5 ± 0.2
Gender						
Men	645	17.9 ± 0.6	512	14.2 ± 0.6	98	2.7 ± 0.3
Women	1695	36.9 ± 0.7	385	8.4 ± 0.4	62	1.3 ± 0.2
Age group (years)						
18-39	1438	44.7 ± 0.8	212	6.6 ± 0.4	7	0.2 ± 0.1
40-49	495	28.1 ± 1.1	205	11.6 ± 0.8	30	1.7 ± 0.3
50-59	268	17.0 ± 0.9	221	14.0 ± 0.9	42	2.7 ± 0.4
60-	139	8.5 ± 0.7	259	15.9 ± 0.9	81	5.0 ± 0.5
Ethnic group						
Han	1662	28.7 ± 0.6	695	12.0 ± 0.4	112	1.9 ± 0.2
Korean	195	20.8 ± 1.3	109	11.6 ± 1.0	34	3.6 ± 0.6
Mongolian	395	31.9 ± 1.3	63	5.1 ± 0.6	10	0.8 ± 0.3
Other	88	38.8 ± 3.2	30	13.2 ± 2.2	4	1.7 ± 0.8
Education						
Primary or below	119	14.5 ± 1.2	100	12.2 ± 1.1	69	8.4 ± 1.0
Secondary	874	24.3 ± 0.7	461	12.8 ± 0.6	48	1.3 ± 0.2
Matriculation or above	1307	36.1 ± 0.8	320	8.8 ± 0.5	42	1.2 ± 0.2
Marital status						
Married	1471	25.1 ± 0.6	724	12.4 ± 0.4	132	2.3 ± 0.2
Single	677	50.9 ± 1.3	51	3.8 ± 0.5	3	0.2 ± 0.1
Divorced/widowed	85	19.3 ± 1.9	51	11.6 ± 1.5	14	3.2 ± 0.8
Smoking						
Non-smoker	1886	32.2 ± 0.6	561	9.6 ± 0.4	110	1.9 ± 0.2
Current smoker	357	20.1 ± 0.9	242	13.6 ± 0.8	35	2.0 ± 0.3
Ex-smoker	49	15.0 ± 2.0	55	16.8 ± 2.1	13	4.0 ± 1.1
Alcohol consumption						
Non-drinker	1825	32.6 ± 0.6	540	9.6 ± 0.4	93	1.7 ± 0.2
Current drinker	413	19.4 ± 0.9	290	13.6 ± 0.7	57	2.7 ± 0.3
Ex-drinker	38	19.5 ± 2.8	24	12.3 ± 2.3	8	4.1 ± 1.4

^*1*^Percent prevalence ± standard error.

^*2*^Abbreviations: PreHTN, prehypertension; PreDM, prediabetes; HPT, hypertension; DM, diabetes mellitus.

### Cardiometabolic risk profile

A summary of the cardiometabolic risk factors of the study population are presented in Table 3, according to their cardiometabolic status (i.e. normotensive and normoglycemic, prehypertensive and prediabetic, or hypertensive and diabetic). SBP (108.9 ± 7.2, 126.3 ± 6.9, 148.9 ± 15.6 mmHg, respectively) and DBP (70.1 ± 6.0, 80.4 ± 5.7, 91.8 ± 10.1 mmHg, respectively; *P *< 0.0001) increased gradually across these categories. Furthermore, there was a steady increase in BMI and WC in both men and women with increasing BP and impaired glucose metabolism. Compared to the normotensive and normoglycemic group, the co-PreHTN and PreDM group had a significantly higher GGT (28.6 [18.0-48.9] U/L), UA (333.4 ± 86.4 μmol/L), and fasting serum glucose (6.0 ± 0.3 mmol/L; *P *< 0.0001). With respect to the fasting lipid profiles, both TG and TC were significantly lower in normotensive and normoglycemic individuals compared to those with co-PreHTN and PreDM (both *P *< 0.0001). Additionally, atherogenic LDL-C was significantly lower, whereas anti-atherogenic HDL-C was significantly higher (both *P *< 0.0001) in normotensive and normoglycemic individuals versus those with co-PreHTN and PreDM.

**Table 3:** A comparison of select age-adjusted cardiovascular risk factors in individuals with normotension and normoglycemia, prehypertension and prediabetes, and hypertension and diabetes

	Normotension-normoglycemia		Prehypertension-prediabetes		Hypertension-diabetes			
			
	N	$\bar{x}\pm s/M({\text{Q}}_{\text{R}})$	N	$\bar{x}\pm s/M({\text{Q}}_{\text{R}})$	N	$\bar{x}\pm s/M({\text{Q}}_{\text{R}})$	*P-value*^*a*^	*P-value*^*b*^
BMI (kg/m^2^)								
Men	645	22.5 ± 3.2	512	25.4 ± 3.5	96	26.6 ± 3.1	< 0.0001	0.0018
Women	1692	22.2 ± 3.9	384	24.6 ± 3.2	61	26.8 ± 3.7	< 0.0001	< 0.0001
WC (cm)								
Men	628	79.0 ± 9.7	498	88.1 ± 9.3	97	92.2 ± 8.5	< 0.0001	< 0.0001
Women	1654	73.3 ± 8.3	376	82.0 ± 8.8	60	88.7 ± 9.8	< 0.0001	< 0.0001
SBP (mmHg)	2430	108.9 ± 7.2	897	126.3 ± 6.9	160	148.9 ± 15.6	< 0.0001	< 0.0001
DBP (mmHg)	2430	70.1 ± 6.0	897	80.4 ± 5.7	160	91.8 ± 10.1	< 0.0001	< 0.0001
GGT (U/L)	2339	15.4 (12.0-22.9)	897	28.6(18.0-48.9)	160	38.1(25.0-64.0)	< 0.0001	< 0.0001
UA (μmol/L)	2339	274.9 ± 74.6	897	333.4 ± 86.4	160	321.3 ± 78.3	< 0.0001	0.0984
GLU (mmol/L)	2340	5.0 ± 0.4	897	6.0 ± 0.3	160	9.1 ± 2.5	< 0.0001	< 0.0001
TG (mmol/L)	2340	0.97(0.73-1.35)	897	1.58(1.08-2.35)	160	2.22(1.52-3.25)	< 0.0001	< 0.0001
TC (mmol/L)	2340	4.4 ± 0.9	897	5.0 ± 1.1	160	5.3 ± 1.1	< 0.0001	0.0015
HDL-C (mmol/L)	2334	1.5 ± 0.3	895	1.4 ± 0.4	160	1.3 ± 0.3	< 0.0001	0.0026
LDL-C (mmol/L)	2340	2.3 ± 0.8	897	2.8 ± 0.9	160	3.1 ± 0.9	< 0.0001	0.0001

^*1*^GGT and TG were reported as medians (interquartile range).

^*2*^Age-adjusted by analysis of covariance.

^*3*^Abbreviations: BMI, body mass index; WC, waist circumference; SBP, systolic blood pressure; DBP, diastolic blood pressure; GGT, γ- glutamyltransferase; UA, uric acid; GLU, glucose; TG, triglyceride; TC, total cholesterol; HDL-C, high density lipoprotein cholesterol; LDL-C, low density lipoprotein cholesterol.

^a ^Normotension-normoglycemia *vs*. prehypertension-prediabetes

^b ^Prehypertension-prediabetes *vs*. hypertension-diabetes

Table 4 demonstrates the correlations between BMI, BP, GLU, and lipid profiles with GGT and UA in individuals with co-PreHTN and PreDM. A multivariate correlation analysis demonstrated that GGT and UA levels were significantly and positively associated with BMI, WC, and SBP in the subjects with co-PreHTN and PreDM. Additionally, a significant and positive correlation was observed between serum TG and GGT/UA. Interestingly, a negative correlation was observed between both GGT and UA with HDL-C. A significant positive correlation was also observed between TC and GGT/UA.

**Table 4:** Correlations between various relevant factors and serum GGT and UA in prehypertensive and prediabetic individuals

Parameters	GGT		UA	
	
	r	*p*	r	*p*
BMI	0.375	0.000	0.289	0.000
WC	0.434	0.000	0.405	0.000
SBP	0.041	0.215	0.074	0.026
DBP	0.148	0.000	0.071	0.333
GLU	0.169	0.000	0.041	0.215
TG	0.454	0.000	0.316	0.000
TC	0.251	0.000	0.139	0.000
HDL-C	-0.206	0.000	-0.236	0.000
LDL-C	0.167	0.000	0.047	0.157

Abbreviations: GGT, γ- glutamyltransferase; UA, uric acid; BMI, body mass index; WC, waist circumference; SBP, systolic blood pressure; DBP, diastolic blood pressure; GLU, glucose; TG, triglyceride; TC, total cholesterol; HDL-C, high density lipoprotein cholesterol; LDL-C, low density lipoprotein cholesterol.

## Discussion

In the present study, the population studied presents a high prevalence of co-PreHTN and PreDM in apparently healthy Chinese adults. The prevalence of co-PreHTN and PreDM was even higher in men than women, and increased with age and BMI. Our findings suggest that prevalence of co-PreHTN and PreDM in our population is similar to that observed in a recent survey conducted in a healthy US population [15]. It has been previously reported that PreDM markedly increases CVD risk in prehypertensive individuals [16]. The recent findings of the Strong Heart Study also revealed that in non-diabetic individuals, IFG and PreHTN increases the number of cardiovascular events by 2.06-fold compared to their normotensive and normoglycemic counterparts, with an absolute increase of 5 cardiovascular events per 1000 person years [10].

It has also been previously shown that there are marked ethnic and geographic differences in BP and FBG levels in China [9,17]. Living conditions, habitual diets, and social circumstances in disparate communities also exhibit different characteristics. In the present study, the prevalence of co-PreHTN and PreDM was higher in the Hei Longjiang province of northern China compared to the Inner Mongolian Autonomous Region in northeastern China (13.3% versus 8.8%). There are a number of potential factors that may contribute to these differences. For example, there are more people in the Hei Longjiang province that are overweight or obese, dyslipidemic, and have a higher salt intake [18]. Unfortunately, data on dietary sodium intake were not collected in the present study. Furthermore, based on the regional differences, there may be genetic contributions to the co-PreHTN and PreDM phenotype that should also be considered.

There was a higher prevalence of co-PreHTN and PreDM among individuals that consumed alcohol and smoked. This finding was consistent with earlier studies, which found that excessive alcohol consumption and cigarette smoking increase BP and glucose levels [19,20]. Interestingly, we also found that there was higher prevalence of ex-smokers than current smokers in the co-PreHTN and PreDM group. This observation can be explained by the findings of Inoue K [21], who suggested that the adverse effects associated with the weight gain following cessation of smoking outweigh the benefits. Weight gain is a strong risk factor for CVD, which is also closely associated with a substantial risk for impaired glucose regulation and elevated BP [22].

In the present study, we also observed that, with the exception of HDL-C, the lipid profile and glucose levels demonstrated a gradual upward trend from the normotensive and normoglycemic group, followed by the co-PreHTN and PreDM group, and lastly the hypertensive and diabetic group. In addition to the larger WC and BMI, our findings also suggest that these considerably "healthy" men and women are at an increased risk for CVD. Other biochemical parameters, such as GGT and UA levels, were also relatively higher in subjects with co-PreHTN and PreDM. Recent cross-sectional and longitudinal studies have also noted a relatively independent association between elevated serum GGT levels, and hypertension and diabetes [23,24]. We have also revealed that higher serum GGT levels are positively associated with PreHTN in an otherwise healthy Chinese adult population following various adjustments in the multivariate logistic regression analysis (unpublished observations). In addition, a recent study [25] found that elevated GGT was independently associated with the presence of the IFG, further supporting our conclusion. Similarly, serum UA levels correlated with metabolic abnormalities and CVD [26]. A previous study[27] found that there is a significant association between serum UA levels and the risk of PreHTN. Meanwhile, in the Rancho Bernardo Study, it was suggested that UA may be a useful predictor of type 2 diabetes in older adults with IFG [28]. However, the underlying mechanisms for higher GGT and UA in subjects with co-PreHTN and PreDM are unclear and require further clarification.

A major strength of our study is that it is a population-based study with a good representative sample of the general Chinese population. Additionally, the larger sample size ensures sufficient power in estimating the prevalence of co-PreHTN and PreDM, as well as determining the correlation between co-PreHTN and PreDM, and CVD risk factors. Lastly, the use of standardized protocols and instruments guaranteed a high response rate and excellent quality control.

However, the study also has several limitations. First, oral glucose tolerance test (OGTT) was not performed in subjects with IFG, thereby potentially reducing the accurate diagnosis of diabetes. Since some individuals with normal FBG may have IGT, based on OGTT data, examining FBG alone (i.e. without a 2-h PBG) may result in the underestimating of the prevalence of co-PreHTN and PreDM in healthy Chinese adults. Lastly, the study is based on a cross-sectional survey, which is unable to determine causality or the temporal relationship between CVD risk factors and co-PreHTN and PreDM.

## Conclusions

Our data highlights the high prevalence of co-PreHTN and PreDM in disease-free Chinese adults from northern and northeastern China. With the exception of HDL-C, higher lipid profile levels, as well as GGT, UA, and FBG, in co-PreHTN and PreDM individuals may have important pathophysiological implications. All of these changes have been shown to increase CVD risk, and thereby suggests that there is an urgent need for early detection and appropriate interventions. A healthy lifestyle, such as weight control, increased physical activity, moderate alcohol intake, tobacco cessation, salt reduction, and sufficient consumption of fresh fruits and vegetables could prevent the progression of co-PreHTN and PreDM to overt hypertension and diabetes mellitus. Public health programs are required to improve this situation in the Chinese population.

## Competing interests

The authors declare that they have no competing interests.

## Authors' contributions

JW carried out the experimental design, analysis and interpretation of data, and drafted the manuscript. WHY carried out the experimental design, performed statistical analysis, and drafted the manuscript. LQ conceived the study, participated in its design and coordination, and helped in drafting the manuscript. XQC, XZG, WW, LYX, and XZQ participated in the study and manuscript revision. YHL and HTD were responsible for the acquisition of data and manuscript revision. SMH, CLX, and GJZ participated in the design of the study and performed the statistical analysis. All authors read and approved the final manuscript.

## Pre-publication history

The pre-publication history for this paper can be accessed here:

http://www.biomedcentral.com/1471-2458/11/794/prepub
